# Mississippi Valley Medical Association

**Published:** 1898-10

**Authors:** Henry E. Tuley, John Young Brown

**Affiliations:** Secretary; Louisville, Ky.; President; Louisville, Ky.


### Mississippi Valley Medical Association.

OFFICE OF SECRETARY. LOUISVILLE, KY., Sept. 15, 1898.

DEAR DOCTOR: You are invited to attend the Twenty-Fourth Annual Meeting of the Mississippi Valley Medical Association, which will be held in the Hall of Representatives of the State Capitol, Nashville, Tenn., commencing at 10 a. m. October 11th, and continuining in session until 1 p. m. October 14th.

Reduced fares can be procured by applying at the railway station on starting, for a certificate; this must be presented to the Secretary not later than the second day of the meeting, to be signed by him and by the Special Agent of the Passenger Associations. This certificate will entitle the holder to a one-third return fare.

Ample provision will be made for the speedy registration of members and guests at the capitol, and all are urged to attend to this immediately upon their arrival. The headquarters will be at the Maxwell House.

The following program gives promise that the meeting will be an instructive and interesting one, and it is hoped that you will attend.

HENRY E. TULEY, Secretary. JOHN YOUNG BROWN, President.

---

PROVISIONAL PROGRAM OF THE TWENTY-FOURTH ANNUAL MEETING OF THE MISSISSIPPI VALLEY MEDICAL ASSOCIATION.

FIRST DAY.—TUESDAY, OCTOBER 11, 1898.

MORNING SESSION, 10:00 O'CLOCK.

Address of welcome.
Reports of officers and committees.
Executive business.
President's inaugural address.

PAPERS.

"The Relations of the Gynecologist and the Neurologist." W. H. Humiston, Cleveland, Ohio. Discussion opened by C. H. Hughes, St. Louis, and Jos. Price, Philadelphia, Pa.

"Diagnostic and Therapeutic Uses of Tuberculin." Chas. W. Aitken, Flemingsburg, Ky.

"Immunity." Chas. T. McClintock, Detroit, Mich.

"Hygiene versus Drugs in Pulmonary Tuberculosis." Charles L. Minor, Asheville, N. C.

"Some of the Factors that Predispose to Tuberculosis." L. P. Barbour, Tullahoma, Tenn.

"The Bicycle from the Medical Standpoint." I. N. Love, St. Louis, Mo.

"Therapeutic Value of Marmoreck's Serum." W. L. Baum, Chicago, Ill.

"Tumors of the Parietal Lobe of the Cerebrum." T. A. Davis, Chicago, Ill.

"Unguentum Hydrargiri or Blue Ointment Administered by the Mouth." Albery Bernheim, Paducah, Ky.

AFTERNOON SESSION, 3:00 O'CLOCK.

"Wounds of the Lachrymal Apparatus: Report of Operation for Restoration of Canaliculi Obliterated by Traumatism." Geo. F. Keiper, Lafayette, Ind.

"Mastoiditis: When to Operate and How." Andrew Timberman, Columbus, Ohio.

"Phophylaxis in Diseases of the Nose and Throat." J. Homer Coulter, Chicago, Ill.

"Three Anomalous Cases of Mastoid Disease." J. L. Minor, Memphis, Tenn.

"Report of Holocain as a Local Anesthetic in Ophthalmic Surgery. E. C. Ellett, Memphis, Tenn.

"Incarceration of the Iris Relieved by Eserine: Report of a Case." Frank Trester Smith, Chattanooga, Tenn.

"A Case of Bilateral Glioma of the Retina: Operation, Non-Recurrence in Seventeen Years." A. G. Sinclair, Memphis, Tenn.

"Tonsillitis or Quinsy: Cause and Treatment." J. A. Stucky, Lexington, Ky.

"Remarks on Hydropthalmus, with Report of Two Cases." James Moore Ball, St. Louis, Mo.

"Conservatism in Oral Surgery" Truman W. Brophy, Chicago, Ill.

"Neuralgias Due to Nasal Origin." Edward T. Dickerman, Chicago, Ill.

"Headache as a Symptom in Eye Disease." W. H. Wilder, Chicago, Ill.

---

SECOND DAY.—WEDNESDAY, OCTOBER 12, 1898.

MORNING SESSION, 9:30 O'CLOCK.

Reports of Committees.

Appointment of Nominating Committee.

Address in Medicine, Dr. James T. Whitaker, Cincinnati, O.

PAPERS.

"Complete Inspection of the Rectum by Means of Newer Mechanical Appliances." Thos. Chas. Martin, Cleveland, O.

"The Relationship Between the Genito-Urinary Tract and Rectum: In Operations Upon the Female, Which Should Receive Priority?" John L. Jelks, Memphis, Tenn.

"Rectal Fistula." J. R. Pennington, Chicago, Ill.

"The Surgical Management of Complex Progressive Ischio-Rectal Fistulæ." Leon Straus, St. Louis, Mo.

"Hydrotherapy in Stomach Diseases." Geo. D. Kahlo, Indianapolis, Ind.

"Phases of Toxæmia from Disturbed Metabolism." Thos. Hunt Stucky, Louisville, Ky.

"The Vascular Dermatoneuroses." A. E. Brayton, Indianapolis, Ind.

"A Clinical Report of a Case of Abscess of the Liver." Edwin Frazer Wilson, Columbus, O.

"The Importance of Early Diagnosis in Surgical Cases." J. C. Morfit, St. Louis, Mo.

"Gonangiectomy and Orchidectomy for Hypertrophied Prostate in Old Men." George W. Johnson, Dunning, Ill.

AFTERNOON SESSION, 3:00 O'CLOCK.

"Why I Have Abandoned the General Practice of Vaginal Hysterectomy." B. Sherwood Dunn, Boston, Mass.

"Why I Do Vaginal Ablation in Pus Cases." Wm. R. Pryor, New York City.

"A Consideration of the Limit to Operative Gynecology." Shelby C. Carson, Greensboro, Ala.

"The Limits of Operations for Cancer of the Uterus." S. S. McMurty, Louisville, Ky.

"Cancer of the Uterus." Louis Frank, Louisville, Ky.

"Surgical Treatment of Pus in the Pelvis." Joseph Price, Philadelphia, Pa.

"Some Pathological Conditions of the Ovaries Causing Pain." G. W. Halley, Kansas City, Mo.

"A Case of Abdominal Hysterectomy With Stercoraceous Vomiting; Recovery." H. Hatch, Quincy, Ill.

"A Plea for Pelvic Cellulitis and Peritonitis." F. F. Bryan, Georgetown, Ky.

"The Diagnosis of Gonorrhea in Women." J. Rilus Eastman, Indianapolis, Ind.

"Care and Repair of the Female Perineum." E. L. Larkins, Terre Haute, Ind.

"Clinical Contribution to Ectopic Gestation." W. W. Taylor, Memphis, Tenn.

"Retro-Displacements of the Uterus and Their Treatment." A. Morgan Cartledge, Louis, Ky.

EVENING.

General reception, Maxwell House.

---

THIRD DAY.—THURSDAY, OCTOBER 13, 1888.

MORNING SESSION.

Reports of Committees.

Address in Surgery. Dr. Geo. Ben Johnson, Richmond, Va.

PAPERS.

"Observations on Surgery of the Kidney." Charles A. L. Reed, Cincinnati, O.

"Direct Diagnosis of Diphtheria." William K. Jaques, Chi cago, Ill.

"Diphtheria and Its Logical Treatment." A. M. Osness, Dayton, O.

"Suprapubic Cystotomy versus Perineal Section." James M. Parrott, Kingston, N. C.

"When Shall we Operate for Appendicitis." Edwin Walker, Evansville, Ind.

"Some Clinical Phases of Intestinal Obstruction." A. H. Cordier, Kansas City, Mo.

"Practical Side of the Treatment of Gunshot Wounds of the Abdomen." H. Horace Grant, Louisville, Ky.

"Surgical Treatment of Ophthalmic Goitre." Bayard Holmes, Chicago, Ill.

"Some Forms of Gangrene and Their Treatment." J. S. Nowlin, Shelbyville, Tenn.

"Sub-periosteal Removal of Caries from the Pelvic Basin, with the Report of a Case." S. E. Milliken, Dallas, Texas.

"Surgical Treatment of Infantile Paralysis." Alex. C. Wiener, Chicago, Ill.

"Interesting Surgical Cases." M. Goltman, Memphis, Tenn.

AFTERNOON SESSION, 3:00 O'CLOCK.

"Neurasthenia and its Treatment." H. C. Sharp, Jeffersonville, Ind.

"A Unique Case of Hernia: Operation." Spencer Graves, St. Louis, Mo.

"Some More About Drainage." Arch Dixon, Henderson, Ky.

"The Triple Operation for Pyloric Stenosis." N. Stone Scott, Cleveland, O.

"Report of a Case of Obstetrics with Complications." R. C. Pratt, McKenzie, Tenn.

"Pichi." H. W. Whitaker, Columbus, O.

"A Few Practical Points in the Treatment of Posterior Urethritis." A. Ravogli, Cincinnati, O.

"Varicocele." F. E. Kelley, LaMoille, Ill.

"Syphilis." John M. Batten, Pittsburg, Pa.

"Prevention of Venereal Disease." David Lieberthal, Chicago, Ill.

---

FOURTH DAY.—FRIDAY, OCTOBER 14, 1898.

MORNING SESSION, 9:30 O'CLOCK.

Report of committee on nominations.

Installation of officers-elect.

PAPERS.

"The Neuro-Hypothesis of Rheumatoid Arthritis." Frank Parsons Norbury, Jacksonville, Ill.

"Intermingling and Change of Type in Diseases." W. Gaston McFadden, Shelbyville, Ind.

"Mercury: Its Action." William F. Barclay, Pittsburg, Pa.

"How Should We Treat Typhoid Fever." T. Virgil Hubbard, Atlanta, Ga.

"The Arthritic Diathesis." R. A. Bate, Louisville, Ky.

"A Triology of Diseases: Acute Articular, Rheumatism, Endocarditis, Chorea." Albert E. Sterne, Indianapolis, Ind.

"Cardiac Murmurs." S. W. Fain, Chattanooga, Tenn.

"The Artificial Production of the Plasmodium Malaria and the Rational Treatment for the Removal of Same in Malaria." L. H. Warner, Brooklyn, N. Y.

"Opium in the Treatment of Epilepsy." Frank C. Hoyt, Chicago, Ill.

Papers have been promised by the following gentlemen, their titles not being received early enough to be inserted in the preliminary program: E. S. Pettyjohn, Alma, Mich.; F. F. Lawrence, Columbus, O.; W. B. Burns, Deckerville, Ark.; H. O. Pantzer, Indianapolis, Ind.; Albert Bernheim, Paducah, Ky., Oscar Dood, Chicago, Ill.; A. E. Halstead, Chicago, Ill.

The session will be held in the hall of the House of Representatives, and the exhibit hall will be the Senate Chamber. Headquarters will be the Maxwell House.

All papers should be typewritten and handed the secretary after reading, for publication in the volume of transactions.

